# Escape from the Darkness: The Need to Provide Elder-friendly Care Policies in Iran

**Published:** 2018-06

**Authors:** Morteza ARAB-ZOZANI, Mahtab ALIZADEH KHOEI, Ali JANATI

**Affiliations:** 1. Iranian Center of Excellence in Health Management, School of Management and Medical Informatics, Tabriz University of Medical Sciences, Tabriz, Iran; 2. Elderly Health Research Center, Endocrinology and Metabolism Population Sciences Institute, Tehran University of Medical Sciences, Tehran, Iran

## Dear Editor-in-Chief

The bell sounds from many years ago. We must plan for the future when the number of births has increased in the early 1980s. During the years, the population ages 65 and above has reached the highest value in Iran in 2015 (1, 2). Population aging associated with many consequences. The risk of chronic diseases and longer care will increase in elderly people at the end of life (3).

One of the key challenges in access to universal health coverage is high burden of diseases and lack of access to sufficient funds (4). In accordance with these challenges pay attention to the population trend is very important because the aging population increases the burden of diseases and subsequently further use of the services.

In the whole world, health systems incurred a high percentage of costs and a greater share of resources allocated (5, 6). Therefore considering long-term policies and planning is of utmost importance. With respect to the existing facilities in the country and the growing trend in population aging, it turns out that the existing facilities will not meet the needs of elderly people in the near future. Therefore, the analysis of past policies and trends can be an important tool for decision-making and forecasting.

Based on raised issues and considering the importance of aging, the authors of this paper intend to conduct a doctoral thesis with the aim of policy analysis to identify the basic needs of elderly and anticipate and prioritize the aged care services in Iran. Because the proper planning leads to better resource allocation and increases client satisfaction and service provider, results of this study can be helpful for decision-making process in providing effective and available aged care services in Iran.
